# Validation of the howRu and howRwe questionnaires at the individual patient level

**DOI:** 10.1186/s12913-015-1093-8

**Published:** 2015-10-02

**Authors:** Steven H. Hendriks, Jojanneke Rutgers, Peter R. van Dijk, Klaas H. Groenier, Henk J. G. Bilo, Nanne Kleefstra, Janwillem W. H. Kocks, Kornelis J. J. van Hateren, Marco H. Blanker

**Affiliations:** Diabetes Centre, Isala, Zwolle, The Netherlands; Department of General Practice, University of Groningen and University Medical Centre Groningen, Groningen, The Netherlands; Department of Internal Medicine, University of Groningen and University Medical Centre Groningen, Groningen, The Netherlands; Department of Internal Medicine, Isala, Zwolle, The Netherlands; Langerhans Medical Research Group, Zwolle, The Netherlands

## Abstract

**Background:**

The howRu and howRwe are new short questionnaires which are meant to measure health-related quality of life and patient experience. However, validation at the individual patient level has not yet taken place. We aimed to investigate the validity of both questionnaires at the individual patient level.

**Methods:**

In this prospective validation study, patients were asked to complete both questionnaires and comment on their answers in a semi-structured in-depth interview. Based on the transcribed interviews, a panel of 45 general practitioners and 45 patients filled out the questionnaires as they thought the patients had completed them. The questionnaires were considered valid instruments when a reliable and acceptable level of agreement was reached between the patient’s score and the score of a review panel, defined as a concordance correlation coefficient (CCC) of ≥0.70. Bland-Altman plots were also made.

**Results:**

Ninety patients were included. The CCC of the howRu total score of the review panel and patients was 0.80 (95 % CI 0.73 to 0.86). Bland-Altman plots showed a mean difference of −0.96 and the limits of agreement ranged from −2.87 to 0.95. The CCC of the howRwe total score was 0.57 (95 % CI 0.42 to 0.69). The mean difference on the Bland-Altman plots was −0.54 and the limits of agreement ranged from −3.59 to 2.52.

**Conclusions:**

The howRu seems to be a valid questionnaire for measuring health-related quality of life at the individual patient level. We do not advice to use the tested version of the howRwe questionnaire for assessing patient experience at the individual patient level.

**Trial registration:**

The study was registered at clinicaltrials.gov NCT01830803.

Registration date: 5 April 2013.

**Electronic supplementary material:**

The online version of this article (doi:10.1186/s12913-015-1093-8) contains supplementary material, which is available to authorized users.

## Background

Assessing health-related quality of life (HRQoL) and patient experience have become more important in the past decades [1, 2]. To measure these aspects, several questionnaires have been designed [3–5]. However, these questionnaires are often unsuitable for large-scale application in daily care, due to the length, complexity and costs [1, 6, 7]. Furthermore, these questionnaires are usually validated at group level, with the inherent risk that results are not valid for the evaluation of individual patients [8, 9].

Benson *et al.* have developed two short generic questionnaires for measuring HRQoL and patient experience: respectively the ‘howRu’ (how are you today?) and ‘howRwe’ (how are we doing?) [10]. Their purpose was to create simple, quick, inexpensive and user-friendly questionnaires that could be generally applicable in daily practice without training of patients, doctors and researchers.

The howRu questionnaire showed good psychometric properties and results similar to the SF-12 in patients with various long-term conditions [10]. However, both questionnaires have not been validated yet at the individual patient level. Therefore, we aimed to investigate the validity of the howRu and howRwe questionnaires at the individual patient level.

## Methods

### Study design and setting

We conducted a prospective validation study in two general practices in Zwolle, a city with 120,000 inhabitants in the north-east of the Netherlands. These general practices together deliver care to more than 12,000 patients in this city. The study was registered at clinicaltrials.gov (NCT01830803) and approved by the Medical Ethical Committee of the University Medical Centre Groningen.

### Study population

Patients who visited a general practitioner (GP) or practice nurse (PN) in the period from February to May 2013 were invited to participate. We used the following exclusion criteria: age below 18 years, illiteracy, insufficient understanding of the Dutch language, mental impairment or such a visual impairment that the questionnaires could not be read. Patients were invited by telephone or approached in the waiting room. All patients gave written informed consent.

### Questionnaires

The howRu is a generic questionnaire for the measurement of HRQoL, consisting of four items concerning discomfort, distress, disability and dependence (see Additional file 1: Figure S4 ). Each item is rated using four levels ranging from ‘none’, ‘a little’, ‘quite a lot’ to ‘extreme’ and each level is assigned a score on a 0–3 ordinal scale, with ‘extreme’ = 0, ‘quite a lot’ = 1, ‘a little’ = 2, ‘none’ = 3.

The howRwe is a generic questionnaire for the measurement of patient experience. This questionnaire has four items concerning promptness, communication, personal relationship and general satisfaction (see Additional file 2: Figure S5 ). Each item is rated using four levels ranging from ‘excellent’, ‘good’, ‘fair’ to ‘poor’ and each level is assigned a score on a 0–3 ordinal scale, with ‘poor’ = 0, ‘fair’ = 1, ‘good’ = 2, ‘excellent’ = 3. For both questionnaires the distinction between the different response choices is emphasized by the use of different colors and icons based on smileys. The howRu/we total scores are calculated by adding the scores for each item. Consequently, the total score ranges between 0 and 12, with higher scores indicating a better HRQoL or patient experience. The howRu and howRwe questionnaires were translated from English into Dutch by the MAPI Institute, which has particular expertise in linguistic validation of questionnaires. The translation process was performed according to a standardized, internationally recognized linguistic validation procedure of translation and back-translation [11].

### Validation method

We applied the validation method proposed by Van der Molen and Kocks to determine the validity of the howRu and howRwe questionnaires at the individual patient level [12]. In this method an in-depth interview with a patient about a specific topic, for example HRQoL, is considered as the gold standard for reflecting the patient’s real thoughts and feelings concerning this topic. This in-depth interview takes place, after a patient has filled out a questionnaire, which aims to measure this topic. A questionnaire is considered a valid instrument at the individual patient level, when a reliable and acceptable level of agreement is reached between the patient’s score and the score of a review panel consisting of independent clinicians, who complete the same questionnaire based on the transcribed interviews. In addition to this validation method, a review panel consisting of independent patients was added to the validation process in this study.

### Study procedures

Potentially eligible patients were asked to fill out the howRu questionnaire prior to consultation with the health care provider and the howRwe questionnaire directly after consultation. Once a patient had completed both questionnaires, the interviewer (JR) decided whether the patient was eligible for an in-depth interview. This selection was based on a desired distribution of patients. We aimed to include 30 patients with diabetes mellitus (DM), 30 patients with chronic obstructive pulmonary disease (COPD) and 30 patients without these diseases. Furthermore, we aimed to include at most 15 patients with a high score (defined 10–12) on the questionnaires for each patient category, in order to get an optimal distribution of scores on both questionnaires.

During the semi-structured in-depth interview, patients were asked to comment on every separate item of the questionnaires. The interview took place preferably on the day of consultation and otherwise within a week after the appointment.

All interviews were recorded and fully transcribed. Three reviewers (JR, PvD, and SH) independently blinded the interviews and discussed discrepancies whilst working in pairs of two for each interview. For this purpose, all possible references to scores on individual items of the questionnaires were covered with black bars of equal length. As a consequence, the review panel could not read, nor derive the selected answers.

Subsequently, 45 unique combinations of interviews (sets) were randomly created using MATLAB (version R2012b). Each set contained 10 different, transcribed and blinded interviews in a unique order with accompanying patients characteristics (gender and age).

These sets were sent to a review panel consisting of 45 GPs and 45 patients unfamiliar with the participants. We invited GPs for participation by sending a letter to a large number of GPs. The reviewing patients were recruited by a call on the website of the Diabetes Association Netherlands (‘Diabetes Vereniging Nederland’) and by letters in three different general practices.

We asked each panelist to read the set of interviews and consequently fill out both questionnaires as they thought the patients had completed them. Each interview was therefore reviewed and scored by five separate GPs and five patients.

### Statistical analysis

All data were manually entered twice to adjust for typing errors. We compared the patients’ scores to the mean scores of the review panel, both for total scores and individual items. We used Lin’s concordance correlation coefficient (CCC) to estimate the degree of agreement (concordance) between patients’ and reviewers’ scores. The CCC combines “accuracy” (bias correction factor (C_b_)) and “precision” (Pearson correlation coefficient (ρ)) and is suitable for numerical data. By using the CCC a good understanding of the sources of (dis)concordance is obtained [13, 14]. The CCC can range from 0 (no agreement) to 1 (perfect agreement). We predefined a CCC score ≥0.70 on the total scores of the questionnaires as an acceptable level of agreement. The internal consistency of the questionnaires for patients and the review panel was calculated using Cronbach’s alpha (α). To show the agreement between patients’ and review panel’s total scores on both questionnaires, we constructed Bland-Altman plots [15]. In these plots the differences between the scores was plotted against the means of these scores. The mean difference, also called bias or systematic error, was calculated. Furthermore, we calculated the limits of agreement (mean difference ± 1.96 × SD of the differences) of the individual differences between patients’ and review panel’s scores. These limits of agreement are considered as reliable when there is a normal distribution of these differences [15]. Normality was evaluated using QQ-plots. A *p*-value <0.05 was considered statistically significant. SPSS version 21.0 and MedCalc version 12.7.2 were used.

## Results

### Population

**Fig. 1:**
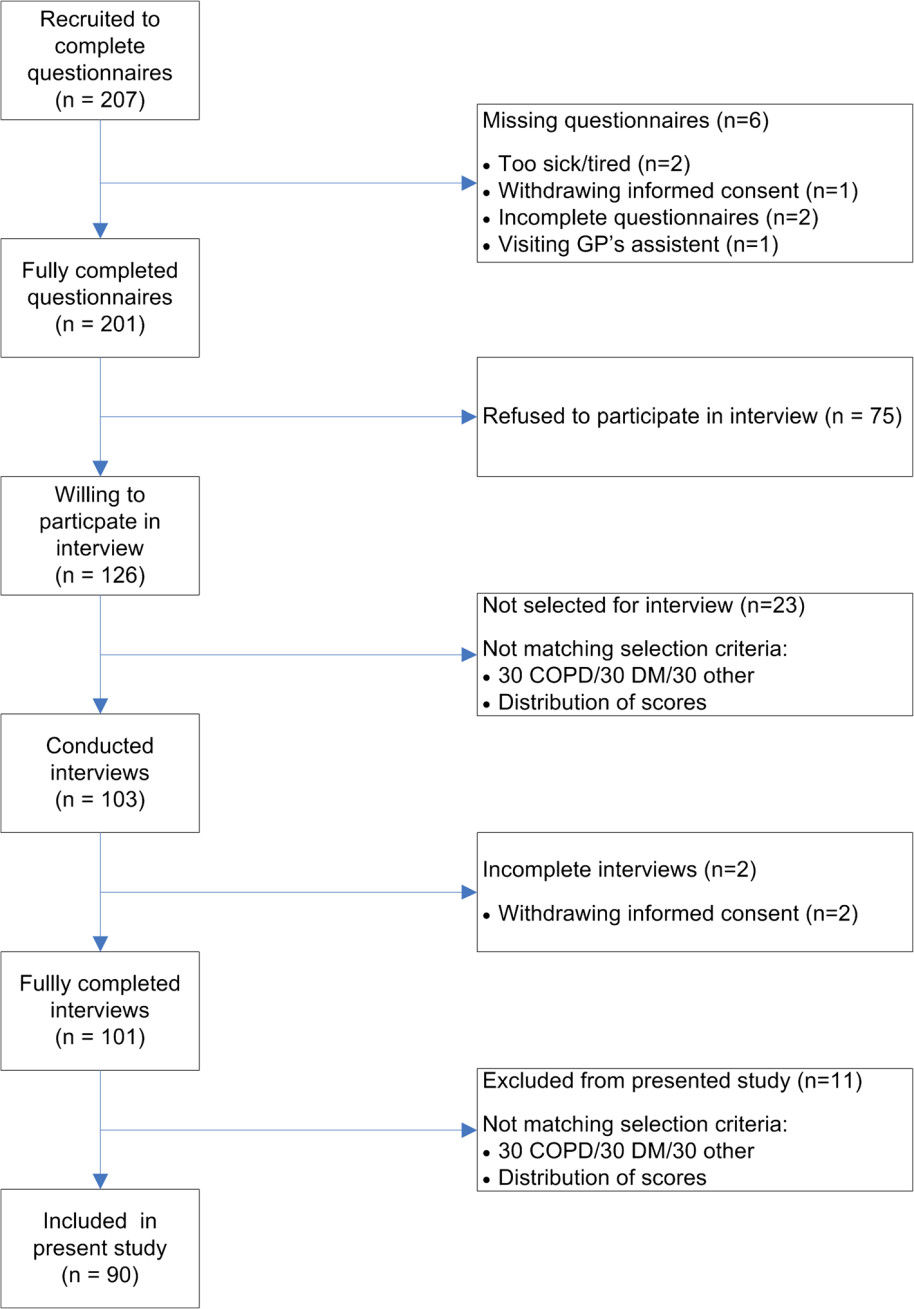
Flow diagram of the selection process

We selected 103 out of 207 patients who filled out the questionnaires for the interview (Fig. 1). Of those, we selected 90 interviews for distribution among the review panel. These patients constitute the basis for this report. Of these, 31 had DM and 10 had COPD, as only few patients with COPD visited the general practices during the study period. Table 1 presents the baseline characteristics of the study population. Most patients (74.4 %) were interviewed on the day of consultation. Thirty-six patients (40 %) had a maximum score, defined as a score of 10–12, on the howRu and 43 of the included patients (47.8 %) achieved a maximum score on the howRwe. On both questionnaires a total score of 4 was the lowest score.

**Table 1:** Baseline characteristics

	Study population (*n = 90*)
Male sex	45 (50)
Age (years)	58 (±16)
Marital status	
Unmarried	11 (12)
Married	60 (67)
Widowed	12 (13)
Divorced	6 (7)
Unknown	1 (1)
Education level	
Low	11 (12)
Average	40 (44)
High	39 (43)
Current smoking	17 (19)
Alcohol consumption	33 (37)
Body mass index (kg/m^2^)	26 (23.2–31.1)
Consultation by GP	57 (63)
HowRu total score	9 (7–10)
HowRwe total score	9 (8–11)

Data are presented as mean (± SD) for normally distributed data and as median with interquartile range for non-normally distributed data or as *n* (%)

### HowRu

The agreement between the patients’ and review panel’s total scores on the howRu, as measured with CCC, was 0.80 (95 % CI 0.73 to 0.86, C_b_ 0.90 and ρ 0.89) (Table 2). For all individual items of the howRu the CCC values were >0.70 (Table 2). Except for item 1, the lower bounds of the 95 % confidence intervals were also >0.70. Accuracy and precision were >0.80 for all items. Cronbach’s α for the howRu items was 0.67 and 0.75 for the patients and the review panel, respectively (data not shown).

**Table 2:** The agreement on the howRu and howRwe questionnaire between patients and the review panel

	Total	Item 1	Item 2	Item 3	Item 4
HowRu		*Pain or discomfort*	*Feel low or worried*	*Limited in what you can do*	*Require help from others*
CCC	0.80 (0.73 – 0.86)	0.72 (0.61 – 0.79)	0.81 (0.73 – 0.86)	0.87 (0.81 – 0.91)	0.86 (0.80 – 0.90)
Accuracy (C_b)_	0.90	0.89	0.92	0.97	0.97
Precision (ρ)	0.89	0.81	0.88	0.89	0.89
HowRwe		*See you promptly*	*Listen and explain*	*Care and respect*	*Meet expectations*
CCC	0.57 (0.42 – 0.69)	0.68 (0.55 – 0.78)	0.50 (0.35 – 0.63)	0.45 (0.28 – 0.59)	0.68 (0.55 – 0.77)
Accuracy (C_b)_	0.95	0.98	0.90	0.93	0.98
Precision (ρ)	0.60	0.69	0.56	0.48	0.69

*CCC* Concordance correlation coefficient

**Fig. 2:**
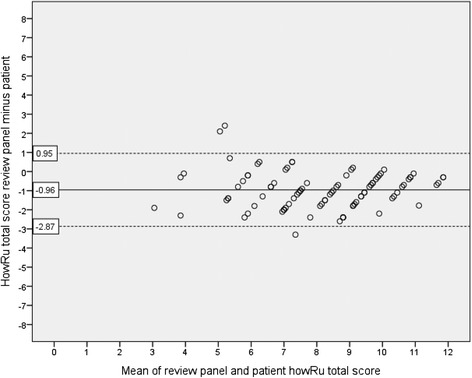
Bland-Altman plot showing the relationship between the howRu total scores of patients and the review panel. The dashed lines represent the limits of agreement

The Bland-Altman plot for howRu total scores is shown in Fig. 2. The mean difference on the total howRu score was −0.96 (95 % CI −1.16 to −0.75), meaning that the review panel scored lower than the patients. This difference was stable over the whole range of scores. The mean differences of all individual items were, as well as the total score, negative on each item (data not shown). The limits of agreement for the howRu total score ranged from −2.87 (95 % CI −3.22 to −2.52) to 0.95 (95 % CI 0.60 to 1.30). The extent of variation in howRu total scores of the individual reviewers is depicted in the Additional file 3: Figure S6.

### HowRwe

The agreement between the patients’ and review panel’s total scores on the howRwe, as measured with CCC, was 0.57 (95 % CI 0.42 to 0.69, C_b_ 0.95 and ρ 0.60) (Table 2). CCC, accuracy and precision for individual items ranged from 0.45 to 0.68, 0.90 to 0.98, and 0.48 to 0.69, respectively. Cronbach’s α for the items of the howRwe was 0.71 for the patients’ and 0.76 for the review panel’s questionnaire (data not shown).

**Fig. 3:**
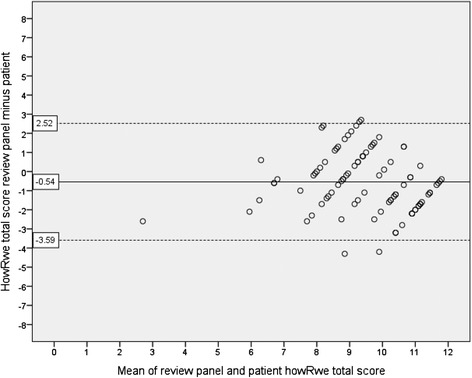
Bland-Altman plot showing the relationship between the howRwe total scores of patients and the review panel. The dashed lines represent the limits of agreement

The Bland-Altman plot for howRwe total scores is shown in Fig. 3. The mean difference on the howRwe total score was −0.54 (95 % CI −0.86 to −0.21), and appeared stable over the whole range of scores. The mean differences of all individual items were, as well as the total score, negative on each item (data not shown). The limits of agreement for the howRwe total score ranged from −3.59 (95 % CI −4.15 to −3.03) to 2.52 (95 % CI 1.96 to 3.08). The extent of variation in howRwe total scores of the individual reviewers is depicted in the Additional file 4: Figure S7.

## Discussion

The Dutch version of the howRu questionnaire seems to be a valid instrument to measure HRQoL at the individual patient level. In our opinion, the howRwe questionnaire is unsuitable for individual measurements of patient experience, because the agreement between patients’ and reviewers’ scores was considered to be too low, and the degree of dispersion too wide.

The developers of the howRu provided support for the validation of the howRu in a study among 2,751 patients with long-term conditions living in the community [10]. They found that the howRu items measure different aspects of an underlying continuum (Cronbach’s α of 0.80) and they also found a high correlation of the howRu with the SF-12. In our study, the Cronbach’s α for the howRu completed by patients was just below the desired interval (0.70 – 0.90). This could be explained by the inclusion of patients with both recent onset conditions and chronic diseases. In addition, the desired number of 30 patients with COPD was not achieved. The missing patients were replaced by others with mostly short-term conditions. As long-term diseases are more likely to affect several areas of HRQoL than recent onset conditions, it is expected that in patients with chronic diseases a greater coherence will exist between different aspects of HRQoL. This will result in a higher internal consistency [16, 17]. The agreement between patients’ and review panel’s howRu scores, as reflected by the CCC, was good. Nevertheless, the Bland-Altman plots showed that the limits of agreement for this questionnaire ranged from −2.87 to 0.95. It is unclear whether this degree of dispersion is acceptable, given the total score range of 0–12. In any ways, it reflects that the review panel tended to score patient HRQoL consequently lower than patients themselves. This may be explained by the fact that patients filled out the questionnaires mostly based upon their first opinion. However, during the in-depth interview they had more time to think about their answers. According to the theory of Daniel Kahneman this could have led to two different opinions with regard to the same subject [18]. Patients might have given a different score, closer to that of the reviewers, if they had filled out the questionnaires after the interview. Nevertheless, in the setting whereby the howRu questionnaire was filled out before the interview, the agreement between patients’ and reviewers’ scores on the howRu was already good enough to validate this questionnaire. However, it might have influenced the results for the howRwe questionnaire.

Concerning the tested version of the howRwe, no official validation studies have been published. In our study, several problems might have influenced the results for this questionnaire. In particular for the howRwe questionnaire, a skewed distribution with many high scores was found. Additionally, the minimum score was only 4, while the range of the questionnaires was 0–12. This could have negatively influenced the CCC value as it is dependent on the heterogeneity within the study sample [19, 20]. However, this skewed distribution is probably inherent to the setting of our study. Generally, patients in primary care in the Netherlands are quite satisfied with the care that is delivered by their GP [21]. Therefore it would be hard to find a wide distribution of the scores in a primary care setting. Finally, the agreement between the patients’ and review panel’s scores was the lowest for the howRwe questions ‘listen and explain’ and ‘care and respect’. These questions have to do with the direct interaction between the caregiver and the patient. The other two questions are partly related to the organization of care. When asking about the interaction between two persons, this is probably more difficult to measure and rate compared with questions which are partly related to the organization of care. Recently, the developers changed two questions of the howRwe with the aim to improve the quality of the questionnaire. The questions ‘care and respect’ and ‘meet expectations’ are changed in ‘treat you kindly’ and ‘well organized’, respectively. This new version of the howRwe showed good psychometric properties and the quality to distinguish between clinical and organizational aspects of patient experience [22]. It has to be studied whether this new version will perform better at the individual patient level.

To our knowledge, this was the second study that applied the validation method of Van der Molen and Kocks to determine the validity of questionnaires at the individual patient level and one can discuss about the suitability of this method for this type of validation [12]. It should be noted that some degree of subjectivity is involved in the blinding of the interviews in this method. However, all interviews have been independently blinded by two different investigators to minimize this subjectivity. Furthermore, we used a mean score for the review panel to reduce the influence of individual reviewers on the results. Nonetheless, it is conceivable that large differences in assessments have been compensated by using averages and that the agreement between the review panel and patients could be actually less.

The simplicity of the howRu makes this questionnaire a good candidate for use in primary care. In comparison with the EQ5D, the howRu is shorter, has a higher completion rate and a smaller ceiling effect [23]. However, relatively little is known about specific psychometric properties of this instrument, such as the minimal clinical important difference (MCID) which indicates clinically relevant differences.

## Conclusions

The results of this study show that based on the CCC the howRu is a valid questionnaire for measuring HRQoL at the individual patient level. However, the wide limits of agreement in absence of an established MCID warrant caution for a too explicit advice. For the further validation of the howRu, research should focus on the questionnaire’s sensitivity to change in comparison with other validated HRQoL questionnaires. We consider the tested version of the howRwe to be unsuitable for assessing patient experience at the individual patient level. Therefore, we believe that this version of the howRwe questionnaire should not be used in daily practice as a single measurement of patient experience. The updated version of the howRwe might perform better, but this assumption has to be studied. Additional research could also focus on the possibility to apply this short and easy questionnaire as a first step in the analyses of patient experience. This would be the case if howRwe scores could predict answers on already validated questionnaires, such as the Europep [5].

## Additional files

Additional file 1: Figure S4. HowRu questionnaire. (TIFF 3820 kb) Additional file 2: Figure S5. HowRwe questionnaire. (TIFF 3970 kb) Additional file 3: Figure S6. The extent of variation in howRu total scores of the individual reviewers. (TIFF 8199 kb) Additional file 4: Figure S7. The extent of variation in howRwe total scores of the individual reviewers. (PNG 56 kb)

## Abbreviations

HRQoL: Health-related quality of life
howRu: How are you?
HowRwe: How are we doing?
GP: General physician
PN: Practice nurse
DM: Diabetes mellitus
COPD: Chronic obstructive pulmonary disease
CCC: Concordance correlation coefficient
C_b_: Bias correction factor
ρ: Pearson correlation coefficient
CI: Confidence interval
