# Supplementary figures and images for: Validation of the howRu and howRwe questionnaires at the individual patient level
Source: BMC Health Serv Res. 2015 Oct 2;15:447. doi: 10.1186/s12913-015-1093-8 (PMC4592573; doi:10.1186/s12913-015-1093-8)

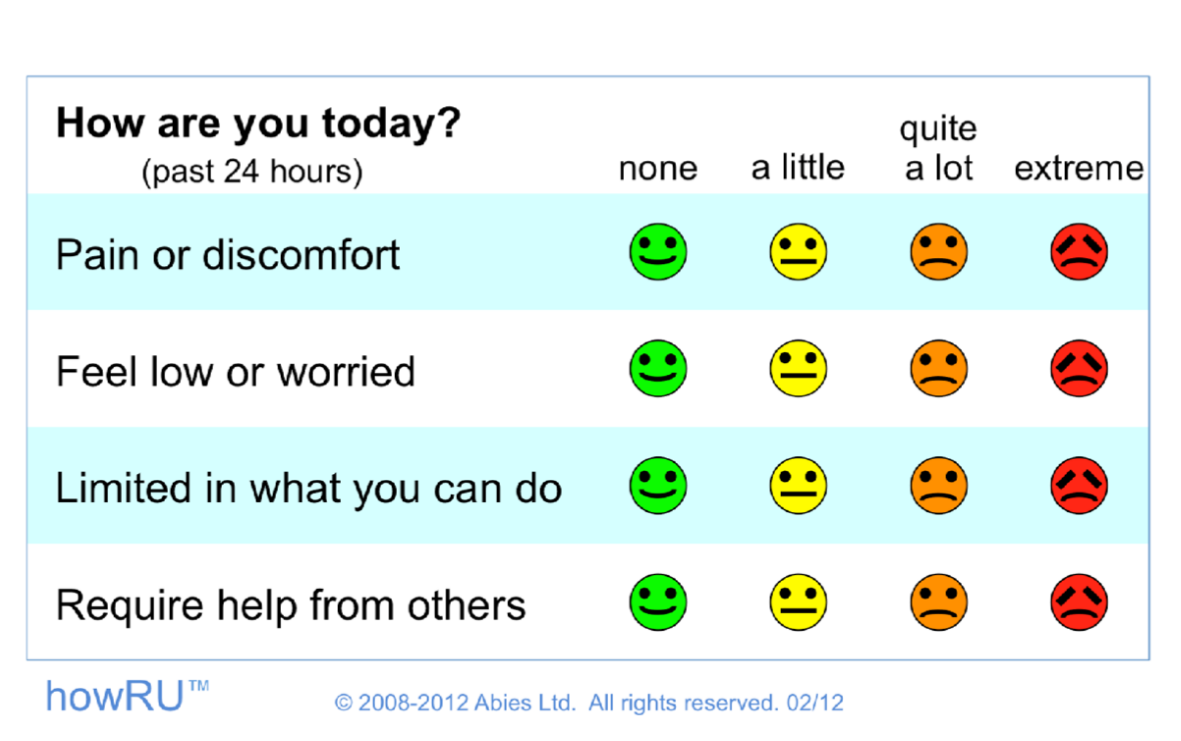

Supplement: Additional file 1: Figure S4. — HowRu questionnaire. (TIFF 3820 kb) [file 12913_2015_1093_MOESM1_ESM.tiff]

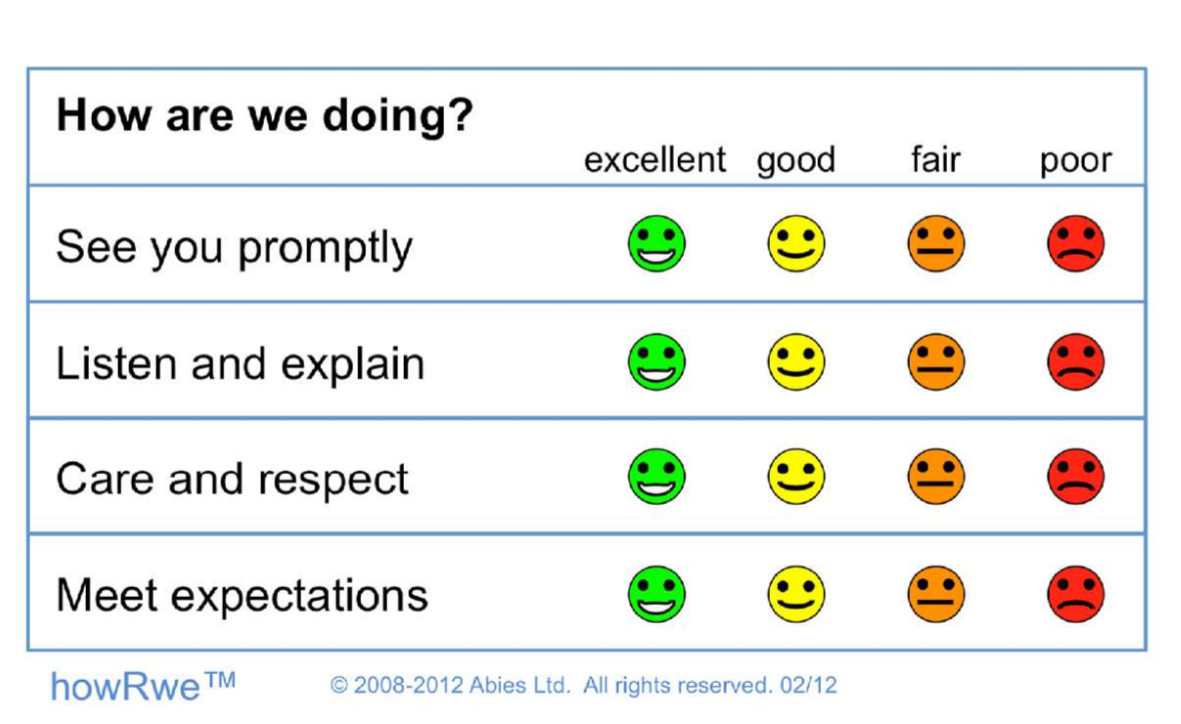

Supplement: Additional file 2: Figure S5. — HowRwe questionnaire. (TIFF 3970 kb) [file 12913_2015_1093_MOESM2_ESM.tiff]

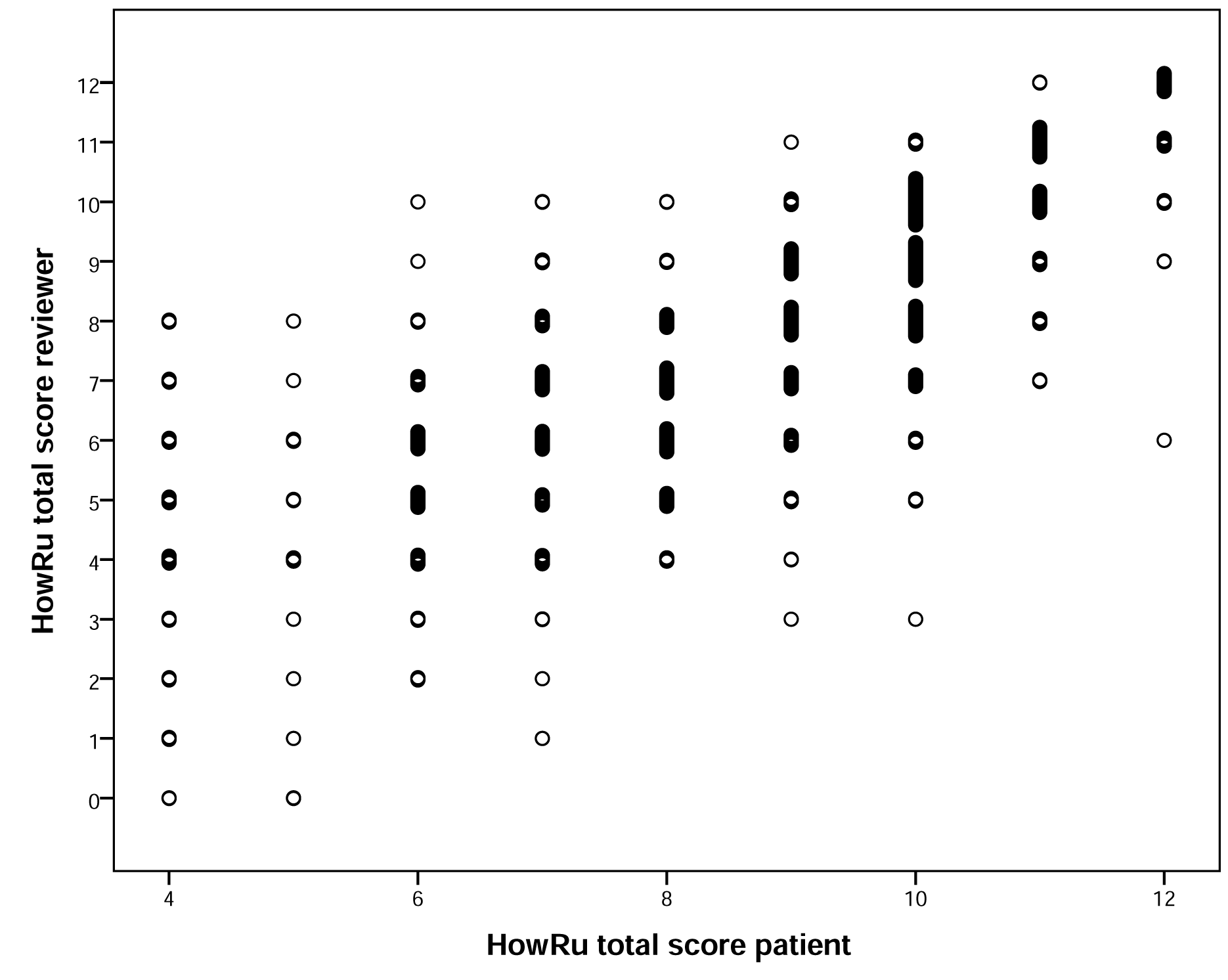

Supplement: Additional file 3: Figure S6. — The extent of variation in howRu total scores of the individual reviewers. (TIFF 8199 kb) [file 12913_2015_1093_MOESM3_ESM.tiff]

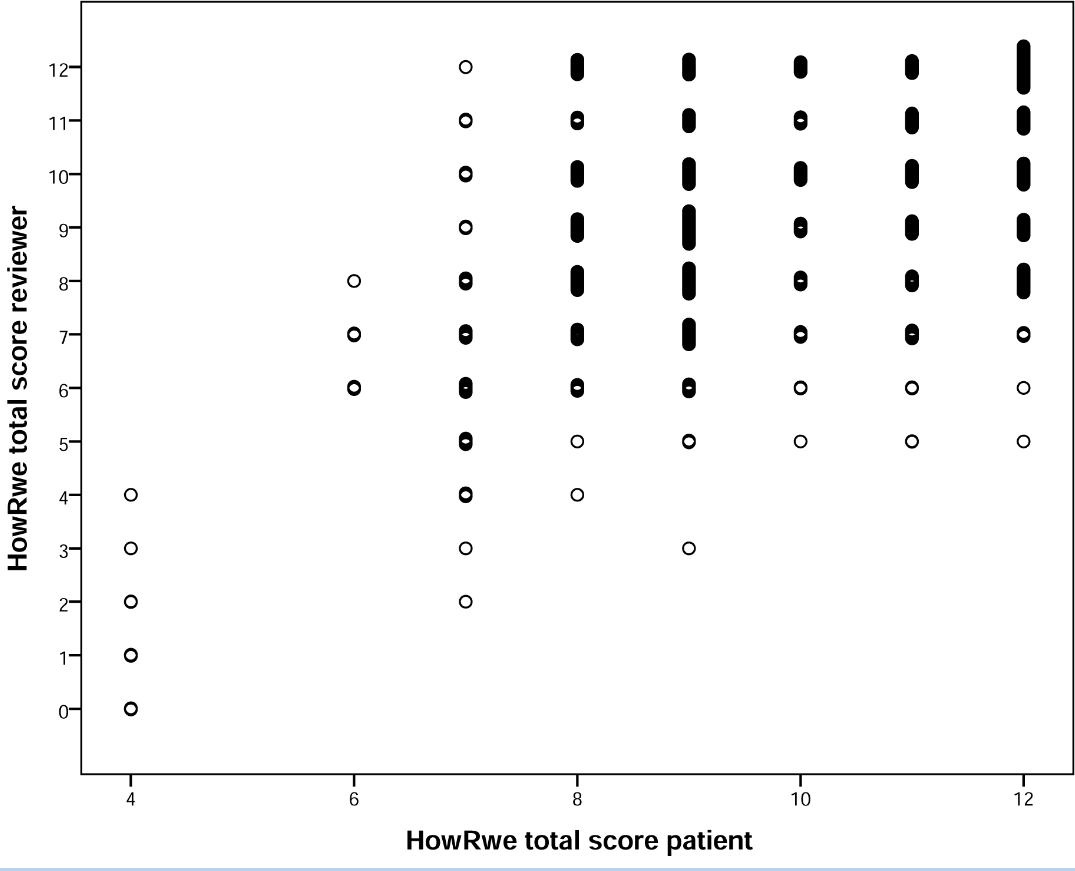

Supplement: Additional file 4: Figure S7. — The extent of variation in howRwe total scores of the individual reviewers. (PNG 56 kb) [file 12913_2015_1093_MOESM4_ESM.png]
